# Plasma Free Fatty Acid Concentration as a Modifiable Risk Factor for Metabolic Disease

**DOI:** 10.3390/nu13082590

**Published:** 2021-07-28

**Authors:** Gregory C. Henderson

**Affiliations:** Department of Nutrition Science, Purdue University, West Lafayette, IN 47907, USA; gchender@purdue.edu

**Keywords:** non-esterified fatty acid, metabolic syndrome, physical activity, caloric restriction, obstructive sleep apnea

## Abstract

Plasma free fatty acid (FFA) concentration is elevated in obesity, insulin resistance (IR), non-alcoholic fatty liver disease (NAFLD), type 2 diabetes (T2D), and related comorbidities such as cardiovascular disease (CVD). Furthermore, experimentally manipulating plasma FFA in the laboratory setting modulates metabolic markers of these disease processes. In this article, evidence is presented indicating that plasma FFA is a disease risk factor. Elevations of plasma FFA can promote ectopic lipid deposition, IR, as well as vascular and cardiac dysfunction. Typically, elevated plasma FFA results from accelerated adipose tissue lipolysis, caused by a high adipose tissue mass, adrenal hormones, or other physiological stressors. Reducing an individual’s postabsorptive and postprandial plasma FFA concentration is expected to improve health. Lifestyle change could provide a significant opportunity for plasma FFA reduction. Various factors can impact plasma FFA concentration, such as chronic restriction of dietary energy intake and weight loss, as well as exercise, sleep quality and quantity, and cigarette smoking. In this review, consideration is given to multiple factors which lead to plasma FFA elevation and subsequent disruption of metabolic health. From considering a variety of medical conditions and lifestyle factors, it becomes clear that plasma FFA concentration is a modifiable risk factor for metabolic disease.

## 1. Introduction

Long-chain fatty acids have a chain length of 12 carbons or greater. The vast majority of stored fatty acids in humans and other animals are in the long-chain form, with the majority being 16 carbons in length or longer and stored through esterification into the triacylglycerol (TAG) pool of white adipose tissue [1,2,3,4]. Alternatively, the fate for short- and medium-chain fatty acids is primarily oxidation [5,6], and thus these fatty acids are not stored as TAG in humans and other animals to an appreciable extent. While TAG is predominantly stored in adipose tissue, it is also stored at ectopic lipid deposition sites such as liver and muscle along with related lipid intermediates. The free fatty acid (FFA) form of fatty acids is an unesterified anion, primarily derived from lipolysis of TAG, and FFA circulate predominantly bound to albumin in the bloodstream [7,8]. In this review, the focus is upon long-chain FFA and, throughout the article, the term ‘FFA’ is used to specifically refer to the long-chain form of this metabolite class, as is a customary nomenclature when studying lipid metabolism [9,10,11,12,13,14]. Circulating FFA provides energy for β-oxidation, and it is believed that this FFA metabolism serves the function of sparing blood glucose through its role as an alternative fuel. In healthy individuals, the ability to rapidly mobilize FFA from TAG storage is potentially important for defense of glycemia during stress such as exercise or fasting [11,15,16,17,18]. However, habitual elevation of plasma FFA beyond energy needs exerts negative health impacts, and plasma FFA elevation is characteristic of metabolic health conditions and unfavorable lifestyle choices. For example, the excess adiposity in obesity leads to elevated FFA in plasma [12,19,20,21,22,23,24,25,26], leading to increased lipid uptake and storage in liver and skeletal muscle. TAG accumulation in liver and muscle, which is a result of excessive plasma FFA level, typically presents alongside elevated levels of lipotoxic intermediates such as diacylglycerol (DAG) [27,28,29,30,31]; DAG and other lipotoxic intermediates (e.g., ceramide) lead to development of insulin resistance (IR) [28,32,33]. To address a proximal step in the development of metabolic dysfunction, reducing the plasma FFA concentration could be desirable. The lipid accumulation in the liver, resulting from excessive plasma FFA, is of particular concern as NAFLD is the most common liver disease in the United States and across the globe and can progress to non-alcoholic steatohepatitis (NASH) and ultimately cirrhosis [34,35]. Furthermore, both hepatic and skeletal muscle lipid accumulation are associated with IR and type 2 diabetes (T2D) risk [33,36,37]. In this review, consideration is given to multiple factors which lead to plasma FFA elevation and subsequent disruption of metabolic health, such as obesity, physical inactivity, obstructive sleep apnea (OSA), sleep deprivation, and cigarette smoking. Each of these health conditions is modifiable to a significant extent. While it has been accepted that plasma FFA concentration plays a role in IR and NAFLD, this comprehensive review article addresses a broader spectrum of factors that are associated with elevation of plasma FFA concentration. When one considers various lifestyle and medical factors, it ultimately becomes clear that there are various modifiable factors, which go far beyond direct effects of adiposity, which can likely exert their negative health effects through an elevation of plasma FFA. To accept plasma FFA concentration as an important modifiable risk factor for disease, the array of modifiable lifestyle factors that impact plasma FFA must first be acknowledged; outlining this association between plasma FFA with lifestyle factors and medical conditions is a goal of this review article. The premise for a role of plasma FFA in disease is further strengthened by appreciating the physiological effects of FFA elevation upon insulin resistance and vascular dysfunction, as discussed in this article.

There are clear indications that reducing plasma FFA chronically could reduce disease burden, and thus significant efforts have been made to develop pharmaceuticals to reduce plasma FFA. These efforts are ongoing but have not yet led to an approved drug aimed at suppressing plasma FFA concentration [38,39]. However, compounds are currently available to temporarily modulate adipose tissue lipolysis to reduce plasma FFA concentration, resulting in short-term improvement in metabolic health; niacin, and its mimetics such as Acipimox, can achieve this result in the short-term, but efficacy of the compounds is not maintained over many months of treatment, and plasma FFA rebounds upward as efficacy is lost. As discussed in other review articles, a search for additional niacin mimetics has led to discovery of compounds that may show a more sustained lipolysis inhibition, yet these compounds have not proceeded onward to approval and clinical use at this time [38,39]. In the present article, the focus is upon describing plasma FFA as a risk factor that can be managed by lifestyle factor such as dietary energy restriction, weight loss, exercise, improved sleep, and abstinence from cigarette smoking. The information presented here on this topic may be considered as examples from biology and medical science that depict the plasticity of plasma FFA concentration and the benefits of reducing it. It is anticipated that this information would provide further justification for the search for pharmaceutical compounds to reduce the plasma FFA level. However, at this point, lifestyle modification is the best tool for long-term modification of plasma FFA concentration.

## 2. Plasma FFA Concentration as a Risk Factor for Metabolic Disease

In people afflicted with metabolic disease, it is common to observe a clustering of various aspects of metabolic dysfunction within the same individual; the term ‘metabolic syndrome’ refers to this phenomenon, in which abdominal obesity, IR, hypertension, and other aspects of T2D and CVD risk coincide [40]. The elevation of plasma FFA that occurs in obesity [12,19,20,21,22,23,24], particularly in abdominal or upper body obesity [12,41], is likely a central factor that explains a significant portion of the metabolic syndrome etiology. That is to say, IR, T2D, NAFLD, and vascular dysfunction are linked through FFA metabolism. Plasma FFA concentration is significantly elevated in people with IR [24,42,43,44] and T2D [21,44,45]. Even in apparently healthy people, higher levels of fasting plasma FFA are predictive of future development of IR [46] and T2D [47], exemplifying that risk for metabolic disease development is worsened by high plasma FFA content. Elevated plasma FFA is also associated with NAFLD [19,20], hypertension [43,44], and poor myocardial function [12,48]. Higher fasting plasma FFA is also associated with elevated risk for all-cause mortality [43,44] and death from CVD [43,44]. In addition to obesity and its associated FFA levels, it is known that various aspects of the metabolic syndrome (IR and vascular health indicators) are detrimentally impacted by factors such as sleep deprivation [49,50,51,52], sleep apnea [49,53,54,55], cigarette smoking [56,57,58,59], and physical inactivity [60,61,62,63]; as discussed below, each of these factors leads to elevated plasma FFA as well, and this impact on circulating FFA concentration may be a causal factor in the detrimental health impacts of these factors. When we take this information as a whole, it becomes apparent that plasma FFA concentration is a disease risk factor which is modifiable.

Evidence indicating plasma FFA concentration as a risk factor for disease is derived from observation of patients (presented above), as well as from laboratory research linking an experimental elevation of plasma FFA with indicators of worsened metabolic health. A common approach for experimentally elevating plasma FFA is to simultaneously infuse heparin and a lipid emulsion (e.g., intralipid); the FFA elevation achieved by heparin and lipid infusion leads to rapid induction of IR [64,65,66,67,68,69,70], ectopic lipid deposition (e.g., in skeletal muscle and liver) [68,69,70,71], endothelial dysfunction [72,73,74], elevated blood pressure [73,74], worsened aortic stiffness [26], and in a T2D population, even increased incidence of cardiac arrhythmia during the infusion [75]. Elevating plasma FFA through use of a low-carbohydrate diet has also been used to confirm the association between plasma FFA and aortic stiffness [26]. Conversely, a short-term reduction in plasma FFA through administration of high-dose niacin (a lipolysis inhibitor), or a niacin mimetic such as Acipimox, reduces plasma FFA [75,76,77,78] and thus reduces ectopic lipid accumulation in the liver [76,77,78], improves markers of insulin sensitivity [75,79,80,81,82], and decreases cardiovascular dysfunction such as cardiac arrhythmia [75]. Additional support for a mechanistic link between plasma FFA concentration and metabolic health comes from work on animal models; adipose triglyceride lipase (ATGL) is the primary controller of lipolysis, and adipose tissue-specific knockout of this enzyme in mice leads to reduced plasma FFA concentration, resulting in improved insulin sensitivity and reduced hepatic lipid content [83,84]. Furthermore, adipose-specific ATGL knockout mice are protected from cardiac pathology in an experimental model of heart failure [85], and this result is also likely to be a result of the reduced plasma FFA concentration. In summary, from observational research and experimental research, it becomes clear that a causal link exists between plasma FFA abundance and metabolic health.

## 3. Modulation of Plasma FFA Concentration by Clinical Interventions and Lifestyle Factors

### 3.1. Body Weight and Energy Balance

The detriments of chronic over-nutrition upon plasma FFA concentration cannot be corrected simply by a single bout of negative energy balance, because acute fasting actually raises plasma FFA concentration and elevates ectopic lipid deposition [15,16,17]. However, the acute effects of fasting should not be discouraging, as chronic caloric restriction (CR) with weight loss eventually appears to reduce plasma FFA. Overweight [26] and obese people [12,19,20,21,24,25] exhibit elevated plasma FFA concentration in the postabsorptive state. Thus, one might be able to assume that sustained weight loss would eventually reduce plasma FFA concentration back near levels of lean individuals. In a study demonstrating higher plasma FFA in obese women compared to lean women, additional insight was obtained by studying women who had experienced significant weight loss following bariatric surgery; the weight-reduced women exhibited lower plasma FFA concentration than the untreated obese women [86]. Next, one can consider findings from numerous studies of weight loss, although the majority have been conducted without employing a weight-stable control group in the study design. For example, in observational studies following gastric bypass surgery patients over time, the association between weight loss and plasma FFA concentration changes has been mixed. In some studies of weight loss from bariatric surgery [87,88,89,90] and CR from dietary intervention [91,92], plasma FFA concentration was reported to be lower after weight loss as compared to plasma from the same individuals before weight loss. In other longitudinal studies (also without weight-stable control groups), this decline of plasma FFA concentration with weight loss from bariatric surgery [25,93] or dietary intervention [93] was not observed. Results are mixed but seem to favor the likelihood of a reduced plasma FFA with weight loss. While these studies are of interest, they were not controlled trials. One should exercise caution when interpreting such findings, as it is difficult to control for effects of various factors that could alter plasma FFA concentration measurements when studies do not include a control group of participants. Randomized controlled trials (RCT) are needed to confirm the apparent findings. Indeed, additional support for the modifiable nature of plasma FFA concentration with weight loss does come from an RCT. The RCT indicated that approximately 6 months after gastric bypass surgery, when substantial weight loss had occurred, patients exhibited lower plasma FFA during an oral glucose tolerance test (OGTT) [94]; thus, the ability to suppress FFA was enhanced by weight loss, although the trend for reduced fasting plasma FFA in this RCT was not statistically significant. Overall, from cross-sectional and longitudinal studies, it generally appears that weight loss acts to reduce plasma FFA concentration, which coincides with the well-accepted health improvements that come from weight loss. However, additional research, particularly from RCT studies, would be helpful to further clarify this conclusion.

### 3.2. Exercise

Even though chronic exercise is known to lead to improved health, a single exercise bout alone is not sufficient to reduce plasma FFA concentration. Indeed, quite the opposite occurs after each individual bout of exercise. As a result of enhanced adipose tissue lipolysis, plasma FFA concentration is elevated during [10,11,95,96,97,98] and for hours after each exercise session [11]. Initially, this information may seem inconsistent with the concept of high plasma FFA concentration being a disease risk factor, as exercise is known to be a behavior with great benefits to health. However, in a physically active individual, many hours per day may be spent following full recovery from exercise. Furthermore, on rest days, habitual exercisers would spend the entire day without exercise-associated stimulation of lipolysis. Thus, we should ask how chronic exercise training impacts plasma FFA, when measurements are made outside of the exercise and recovery time period. This information is needed in order to fully appreciate the role of plasma FFA in health and its potential status as a modifiable risk factor. Initial clues come from an observational study of T2D patients; it was reported that patients who reported higher levels of daily physical activity also had the lowest plasma FFA concentrations amongst the group of study participants [75], indicating a negative statistical association between physical activity level and fasting plasma FFA when measurements of plasma FFA are made outside of the post-exercise recovery time window. Furthermore, a previously published meta analysis addressed this issue by analyzing effects of chronic exercise training upon plasma FFA concentration in overweight, obese, and NAFLD patients; the meta analysis of RCT results revealed a significant reduction in plasma FFA concentration with chronic exercise training [99]. Studies with an RCT study design that have shown a reduction in plasma FFA concentrations with chronic exercise training have employed endurance exercise training at a challenging intensity (70% of VO_2_max or higher) with variable total training volumes [100,101,102]. In exercise RCTs, it is not always reported how many days of rest were allowed before post-training measurements; however, when reported for studies demonstrating reduced plasma FFA with training, the post-training measurements were at least 3 days after the last day of exercise [101,102]. More data are needed, and currently it is unclear whether resistance exercise or a wide variety of endurance exercise approaches lead to reduced plasma FFA concentration. Yet, the results described above provide proof of principle that at least certain types of chronic exercise approaches can reduce plasma FFA concentration [99]. The reduction in plasma FFA that occurs with chronic exercise training corresponds to the well-accepted effect of exercise to reduce hepatic TAG and to improve insulin sensitivity [103,104,105]. As plasma FFA reduction is expected to reduce the severity of hepatic steatosis and IR, there may be a mechanistic role of plasma FFA in the chronic training response.

### 3.3. Sleep Deprivation

Insufficient sleep quantity and quality can lead to IR, heightened T2D risk, and NAFLD [49,50,51,52]. As plasma FFA concentration changes could potentially be a root cause of this effect of sleep dysfunction on disease risk, below the effects of sleep deprivation on plasma FFA concentration are reviewed. As discussed above in relation to weight loss studies, it is important to be cautious when interpreting studies that did not employ a control group. Two studies of sleep deprivation were reported (5 h vs. 10 h of ‘time in bed’ per night) using data from the same study participants [106,107]; participants were studied before, during, and after this reduction in sleep to 5 h per night, yet no control group was studied over the same timeframe. In one of these studies, plasma FFA concentration was surprisingly reduced after four nights of sleep restriction and remained reduced below baseline after restoration of sleeping patterns [107]. In the second study, following an additional night of sleep restriction (5 nights total), plasma FFA actually tended to be elevated, though not to a statistically significant extent [106]. Without knowing the level of drift of plasma FFA concentration over 4–5 days that would have occurred in a control group, it is difficult to interpret the findings from these studies [106,107] conclusively. Thus, next in this section, the focus is upon studies that report investigation of a control condition and a sleep deprivation condition in randomized order. From these controlled studies, it becomes clear that multiple nights of sleep deprivation consistently leads to elevated plasma FFA concentration. Five nights of sleep restriction (4 h compared with 8 h sleep per night) led to increased fasting plasma FFA and induced IR; the participants (a combined group of men and women) also exhibited elevated cortisol and catecholamines in urine in response to sleep deprivation, suggesting a potential role of the hypothalamic–pituitary–adrenal (HPA) axis and sympathetic nervous system activity [52]. In a study that further supported this finding, four nights of sleep restriction in men (4.5 h sleep compared with 8.5 h per night) led to increased plasma FFA during the nighttime and morning, alongside a worsening of their IR; the sleep restricted participants also exhibited elevated plasma cortisol and norepinephrine (lipolytic hormones) at these time points [51]. Furthermore, in this study, the authors demonstrated a significant correlation between elevated nighttime plasma FFA and the reduction in insulin sensitivity on the next day [51]; thus, elevated plasma FFA concentration may have led to the subsequent IR that resulted from sleep deprivation. While multiple nights of sleep deprivation leads to elevated plasma FFA and metabolic dysfunction, it is less clear if a single night of such a stressor would be sufficient to exert this detrimental impact. Complete abstinence from sleep for one day did not alter plasma FFA or glucose [108,109]. In another study, when partial restriction of sleep time was for a single night (4 h vs. 8 h sleep), while the stimulus was not sufficient to raise fasting plasma FFA, it did lead to impaired ability to suppress plasma FFA under insulin-stimulated conditions; this single night of reduced sleep duration resulted in worsened insulin sensitivity and elevated plasma FFA during a hyperinsulinemic euglycemic clamp [110]. Maintaining lower plasma FFA concentrations is desirable for metabolic health maintenance both in the postabsorptive and postprandial states. An impaired ability to suppress plasma FFA in response to insulin indicates a negative impact of sleep deprivation which could potentially be manifested following meals in an individual’s daily life. As a whole, the literature indicates that multiple nights of sequential sleep restriction leads to elevated plasma FFA and impaired glycemic regulation, and this observation is consistent with the known relationship between plasma FFA concentration and insulin resistance.

### 3.4. Sleep Apnea

OSA increases risk for developing IR, other metabolic syndrome factors, and vascular disease [49,53,54,55]. The literature indicates that this type of sleep dysfunction also elevates plasma FFA concentration. The plasma FFA elevation can be corrected through treatments of the sleep apnea, as discussed below, and these findings indicate that circulating FFA abundance is a potential mechanism by which treatment can improve health. In a study of male and female patients with OSA, treatment with continuous positive airway pressure (CPAP) was studied, and CPAP reduced plasma FFA, glucose, and cortisol concentrations [111]; the authors interpreted the finding to suggest that hypoxia and sleep fragmentation in OSA each increase activity of the HPA axis and sympathetic nervous system, while CPAP can correct the abnormality. In another study assessing effects of treatment, male and female congestive heart failure patients were studied; heart failure patients with and without OSA were compared to one another. The patients with OSA exhibited higher plasma FFA during the nighttime sleeping hours, and administering supplemental oxygen to treat OSA reduced plasma FFA to normal levels [112]. In a cross-sectional study comparing patients with and without OSA, it was discovered that fasting plasma FFA and glucose were higher in OSA patients than controls [54]. Furthermore, the severity of OSA in these men and women correlated with the magnitude of plasma FFA elevation [54]. In another cross-sectional study, comparing men with and without OSA, the OSA patients exhibited higher fasting glucose, a worsened glucose excursion during an OGTT, and less suppression of plasma FFA during the OGTT [55]. In conclusion, OSA is a common comorbidity of obesity which leads to worsened metabolic health, likely in part through effects upon plasma FFA concentration. Treating OSA can reduce plasma FFA, indicating that plasma FFA concentration is modifiable as a potential means to improve health.

### 3.5. Cigarette Smoking

When considering the detrimental impacts of cigarette smoking, the focus is typically upon lung cancer and chronic obstructive pulmonary disease (COPD). However, it is important to note that chronic smokers also exhibit IR and elevated T2D risk [56]. In male and female smokers with T2D, during even a single session of cigarette smoking (1 cigarette per hour), plasma FFA concentration was elevated and IR was worsened [113]. Furthermore, in studies of non-diabetic habitual male smokers, plasma FFA concentration was elevated acutely after smoking two cigarettes on the study day [114] and plasma FFA concentration was even elevated following smoking only a single cigarette in another study [115]. In a study comparing chronic smokers with non-smokers, each with T2D, it was shown that diabetic smokers have worse insulin sensitivity than diabetic non-smokers, and the smokers also exhibited a worsened ability to suppress plasma FFA during a hyperinsulinemic euglycemic clamp [58]. In another study of regular smokers, an acute smoking bout (rate of 2 cigarettes per hour) substantially elevated plasma FFA concentration; in this study stable isotope tracers were used to also demonstrate an elevated plasma FFA rate of appearance in response to smoking, indicating most likely that adipose tissue lipolysis was the cause of the elevated plasma FFA during smoking [116]. In further support of these findings, in a study of male smokers compared with non-smokers, plasma FFA was substantially higher in the fasted state and muscle insulin sensitivity was lower in smokers [57]. Thus, overall, this significant body of literature indicates that abstinence from smoking would help people maintain lower plasma FFA levels and thus superior metabolic health. Next, one may wonder if electronic cigarettes or other sources of nicotine would exert similar impacts upon plasma FFA, as any means of delivering nicotine might lead to elevated plasma FFA. From work in mice, it is known that chronic nicotine administration exacerbates the obesity-related elevation of plasma FFA concentration and increases skeletal muscle lipid accumulation [117], and nicotine also increases hepatic lipid accumulation in mice [118]. Furthermore, nicotine administration in mice also decreased the ability to suppress plasma FFA concentration in response to insulin administration in vivo and decreased the suppression of lipolysis in adipose tissue in response to insulin treatment in vitro [118]. In addition to direct effects upon adipose tissue, nicotine appears to stimulate lipolysis through increased epinephrine and norepinephrine secretion, as demonstrated in an intravenous nicotine infusion study in men [119]. Thus, in conclusion, responses to smoking and other forms of nicotine intake provide support for the notion that plasma FFA concentration is a modifiable risk factor for metabolic disease and is responsive to lifestyle factors.

## 4. Mechanisms

Adipose tissue lipolysis is considered to be a primary determinant of the plasma FFA concentration. Thus, any worsening of the ability to restrain lipolysis in adipose tissue could lead to elevated plasma FFA. The elevated adipose tissue mass in obesity is statistically associated with the accelerated release rate of FFA into circulation. While in obesity the rate of FFA release per gram of adipose tissue is somewhat reduced, because total adipose mass is substantially increased in obesity, the net results is that obese people exhibit an elevated total rate of whole body plasma FFA turnover [120]. Therefore, elevated fat mass alone may be sufficient to elevate the plasma FFA abundance. IR in adipose tissue could be another factor that promotes elevated plasma FFA, as insulin exerts potent anti-lipolytic effects in healthy adipose tissue but not in insulin resistant adipose tissue. Adipose tissue IR is typically assessed by the degree of plasma FFA suppression during an OGTT or during a hyperinsulinemic euglycemic clamp. Indeed suppression of plasma FFA concentration by insulin is blunted in T2D [45], as well as in other instances of IR such as sleep deprivation [110] and following cigarette smoking [58,113]; furthermore, weight loss via gastric bypass surgery [94] and treatment of sleep apnea [55] each enhance the ability to suppress plasma FFA concentration during an OGTT. Activation of the sympathetic nervous system can also impose challenges upon metabolic health, as β-adrenergic signaling in adipose tissue enhances lipolysis and thus accelerates the release of FFA into circulation. As discussed above, excessive FFA in circulation can promote IR and vascular pathology, and thus over-activation of the sympathetic nervous system can be problematic. Stress hormones such as catecholamines (epinephrine and norepinephrine) as well as cortisol promote adipose tissue lipolysis and appear to be potentially elevated in some studies when investigating factors such as sleep restriction [51,52] and cigarette smoking [121]. Furthermore, the reduction in plasma FFA in sleep apnea observed during CPAP treatment is associated with a reduction in cortisol levels [111], further indicating that lipolytic control by stress hormones is a potential mechanism for changing plasma FFA concentration with lifestyle and medical treatments.

Following consideration of the physiological mechanisms that typically govern changes in plasma FFA concentration, next it is important to consider the mechanisms that may link those FFA abundances to pathology and disease risk. While the primary focus of this review article is to highlight evidence that plasma FFA concentration is a risk factor for disease at the organismal level, below a brief review is presented of potential cellular mechanisms by which plasma FFA concentration exerts its detrimental effects. Uptake of FFA into cells occurs down concentration gradients. When intracellular FFA concentrations are elevated, FFA can be lipotoxic and lead to cell death and dysfunction [122,123,124]. In addition to TAG storage in lipid droplets, intermediates in the pathway toward lipid storage (e.g., DAG) can accumulate when lipid supply is excessive [27,28,29,30,31]. Elevated DAG can lead to IR though activation of specific protein kinase C isoforms [28,32,33]. Additionally, other intermediates in the TAG synthesis pathway (phosphatidic acid and lysophosphatidic acid) as well as ceramides are potentially lipotoxic [33,125,126,127,128]. NAFLD is associated with IR and is mechanistically linked to cellular steatosis and excessive FFA supply from plasma. The cellular dysfunction in NAFLD can cause progression to NASH when inflammation and related pathological processes are triggered. The steatosis and elevated intracellular FFA in NAFLD can lead to increased production of reactive oxygen species (ROS), due to elevated substrate supply to the fatty acid oxidation pathways [129,130,131]; the steatosis and FFA elevation in liver cells can also lead to endoplasmic reticulum (ER) stress [129,130,131,132]. Oxidative stress and ER stress each contribute to inflammation and downstream induction of apoptosis [129,130,131,132]. In addition to effects of excessive FFA supply upon IR and NAFLD, there are also detrimental effects upon the vasculature and cardiovascular health. The stimulation of ROS production by FFA is a commonality amongst various tissues, including vascular endothelial cells [112,133,134]. FFA elevation can harm vascular health likely through various mechanisms, acting through multiple aspects of the metabolic syndrome and through effects of steatosis in multiple tissues, including in the heart. Nonetheless, it is specifically noteworthy that elevated plasma FFA concentration exerts impacts directly upon the vascular endothelium; FFA elevation leads to endothelial dysfunction, manifested as blunted control of vasodilation [72,133,134,135], potentially caused by ROS-mediated impairment of endothelial nitric oxide synthase activity [112,133,134]. In summary, research on the physiological effects of elevated cellular FFA supply and lipotoxicity indicate a compelling link between plasma FFA elevation and poor health outcomes in people afflicted with obesity, physical inactivity, sleep deprivation, OSA, and in those who use nicotine products such as cigarettes.

## 5. Summary and Conclusions

In conclusion, habitual elevation of plasma FFA concentration in the postabsorptive and postprandial states can lead to heightened risk for developing various diseases related to metabolic syndrome, IR, and cardiovascular function. Plasma FFA concentration should not replace other well-established risk factors for disease (e.g., body mass index, serum low-density lipoprotein and triacylglycerol). Rather, FFA concentration should be potentially added to the panel of clinical factors that are considered as components of risk determination for disease. While there are established management plans for some risk factors such as low-density lipoprotein cholesterol [136], for plasma FFA, additional efforts will be needed to establish harmonized methods for biomarker assessment and guidelines for management. Reducing one’s plasma FFA concentration throughout the day should be a goal. Plasma FFA concentration is modifiable by lifestyle factors, and thus we need not wait for novel drug development to begin acting upon this risk factor. Though initially it had appeared that exercise may simply elevate plasma FFA, it is now apparent that chronic exercise training may actually blunt plasma FFA concentration at times that are away from the exercise session and outside the post-exercise recovery period. Furthermore, it now appears likely that plasma FFA concentration can be reduced by treating obesity with weight loss and by medically treating any associated sleep apnea. Finally, while there have been many valid reasons to abstain from cigarette smoking in the past (e.g., cancer and COPD), additionally it has become apparent that smoking or taking other nicotine products leads to elevated plasma FFA and thus IR. Identifying FFA as a modifiable risk factor for metabolic disease paints the picture of a biological and clinical linkage between various factors that alter metabolic health, such as obesity, physical inactivity, sleep, smoking, and potentially other factors that are not yet appreciated. Work related to target identification and drug development aimed at suppressing plasma FFA is ongoing, yet also actionable information is available now with regard to implementation of lifestyle recommendations for managing one’s plasma FFA concentration.

## Data Availability

Not applicable.
